# Low-intensity blood flow restriction calf muscle training leads to similar functional and structural adaptations than conventional low-load strength training: A randomized controlled trial

**DOI:** 10.1371/journal.pone.0235377

**Published:** 2020-06-30

**Authors:** Simon Gavanda, Eduard Isenmann, Yvonne Schlöder, Roland Roth, Jürgen Freiwald, Thorsten Schiffer, Stephan Geisler, Michael Behringer

**Affiliations:** 1 Department Fitness & Health, IST University of Applied Sciences, Düsseldorf, Germany; 2 Institute of Cardiology and Sports Medicine, German Sport University Cologne, Cologne, Germany; 3 Department of Sport Science Movement and Training Science, University of Wuppertal, Wuppertal, Germany; 4 Outpatient Clinic for Sports Traumatology and Public Health Consultation, German Sport University Cologne, Cologne, Germany; 5 Institute of Sports Sciences, Goethe-Universität Frankfurt am Main, Frankfurt am Main, Germany; Universidade Federal de Mato Grosso do Sul, BRAZIL

## Abstract

The purpose of this study was to investigate whether a six-week, twice weekly resistance training (4 sets at 30% 1-RM until failure) with practical blood flow restriction (BFR) using 7cm wide cuffs with a twist lock placed below the patella is superior to training without BFR (NoBFR) concerning muscle mass and strength gains in calf muscles.

A two-group (BFR n = 12, mean age 27.33 (7.0) years, training experience 7.3 (7.0) years; NoBFR n = 9, mean age 28.9 (7.4) years, training experience 7.1 (6.6) years) randomized matched pair design based on initial 1-RM was used to assess the effects on structural and functional adaptations in healthy males (Perometer calf volume [CV], gastrocnemius muscle thickness using ultrasound [MT], 7-maximal hopping test for leg stiffness [LS], 1-RM smith machine calf raise [1-RM], and visual analogue scale as a measure of pain intensity [VAS]).

The mean number of repetitions completed per training session across the intervention period was higher in the NoBFR group compared to the BFR group (70 (16) vs. 52 (9), p = 0.002). VAS measured during the first session increased similarly in both groups from first to fourth set (p<0.001). No group effects or time×group interactions were found for CV, MT, LS, and 1-RM. However, there were significant time effects for MT (BFR +0.07 cm; NoBFR +0.04; p = 0.008), and 1-RM (BFR +40 kg; NoBFR +34 kg; p<0.001).

LS and CV remained unchanged through training. VAS in both groups were similar, and BFR and NoBFR were equally effective for increasing 1-RM and MT in trained males. However, BFR was more time efficient, due to lesser repetition per training session.

## Introduction

In addition to the gluteal and upper leg muscles, the calf muscles have a crucial impact on the sprint and jumping performance, as they develop most of the final push, that accelerates an athlete upwards and forward. In a recently published study, Möck et al. presented medium to strong correlations between the one-repetition maximum (1-RM) standing calf raises and sprint performance (30m) of sport students [1]. However, it is worth mentioning that for improving sprint performance strength training shows on average only small effect size (ES = -0.32) [2]. Moreover, the literature available so far indicates that calf muscles in general are difficult to train. Previous studies have shown little to no improvement of the calf muscles in terms of hypertrophy and strength gains [3]. A meta-analysis on this topic showed that the effect size for hypertrophy across all studies and training methods (resistance training [RT] and neuromuscular electrical stimulation) was small (Hedges g = 0.30; 95% confidence interval:0.01–0.59; p = 0.05; random effects model) [4]. This is supported by Trappe et al. [3], who reported that calf muscles present with a lower muscle protein synthetic response compared to the vastus lateralis following three different calf muscle exercises that were performed for 4 sets of 15 repetitions at 70% of 1-RM. The underlying mechanisms, however, for this remain unclear.

In order to achieve muscular adaptations in the sense of muscle hypertrophy and strength gains, it was long assumed that moderate to high resistances had to be overcome. However, recent research indicates that mechanical tension through loading is not the only way to achieve these adaptations [5]. An alternative option may be the application of metabolic stress, caused by the accumulation of various metabolites which can be achieved at low intensities with high repetition rates and muscular fatigue. However, this training stimulus occurs much earlier if the blood supply to the working muscles is reduced and venous reflux is prevented. The effectiveness of this training method, known as blood flow restriction training (BFR), has already been demonstrated in countless studies [6]. Meta-analyses indicate that the achievable effects in terms of muscle strength are somewhat lower and the effects of muscle hypertrophy are comparable to what can be expected from high-intensity RT [7].

It can be assumed that, based on previous findings, that the applied training stimulus in the form of classical RT was not optimal for achieving muscular hypertrophy of the calf musculature. Metabolic stress may be more suitable. Research also demonstrates that RT of the calf muscles with BFR is able to increase microvascular filtration capacity [8]. In combination with highly intensive training, BFR training achieves improved fatigue resistance, faster regeneration and improved oxygen consumption during exercise after just one week [9]. However, data on whether low-intensity RT with BFR is able to increase muscle strength and mass is still lacking.

Therefore, the aim of the present study was to investigate whether a six-week low-intensity RT with BFR is superior to RT without BFR for inducing muscle mass and strength gains, when performed to volitional muscle failure.

## Materials and methods

### Design

A two-group randomized matched-pair design, based on initial 1-RM, was used to assess the effects of a six-week low-intensity BFR or low-intensity conventional calf muscle RT (NoBFR) on structural and functional adaptations. The independent variables in this study consisted of two different training modalities (BFR and NoBFR) and six dependent variables: calf volume, gastrocnemius muscle thickness, leg stiffness, and 1-RM calf raise, as well as a pain assessment during the first training session.

Prior to the investigation, ethical clearance was obtained from the IST University of Applied Sciences ethics committee according to the Declaration of Helsinki.

### Subjects

The G*Power software (3.1.9.2, Universität Düsseldorf, Germany) was used in advance to determine necessary sample size via power analysis using a medium effect size (f = 0.25; α = 0.05; 1-β = 0.80) [10]. To account for possible dropouts thirty participants were recruited in a local commercial gym. Inclusion criteria were as follows: male, 18–45 years old, at least two years RT experience, no use of nutritional supplements or illegal drugs (i.e. anabolic steroids), and no existing respiratory, cardiovascular, or musculoskeletal disorders. One subject did not meet medical inclusion criteria and was excluded prior to the study. All participants were informed about the study procedure and methods used, as well as potential risks, and gave written informed consent before the start of the intervention. Subjects agreed to abstain from any additional calf RT for the course of the study. Furthermore, to minimize possible dietary confounding results, subjects were asked to maintain their normal diet throughout the study.

### Methodology

#### Pre-testing

For each subject pre- and post-testing was performed at the same time of day. During pre-testing, subjects’ training history was assessed via questionnaire. Subsequently, body mass was measured using electronic weighing scale (Seca 803; Seca GmbH & Co. KG, Hamburg, Germany) and whole calf volume (CV), as an indirect measure for hypertrophy, was calculated using PeroPlus (Pero-System Messgeräte GmbH, Wuppertal, Germany) from a series of vertical and horizontal diameter measurements taken by an optoelectronic device (Perometer 350 NT; Pero-System Messgeräte GmbH, Wuppertal, Germany) [11]. Same day measurement error has been reported to be 4.3% [11]. Subjects were seated on a chair with the right leg completely extended and relaxed in the middle of the measuring frame, while the foot was positioned on the footrest of the device. The measuring frame was moved proximally along the rail of the device from the most bottom position over the knee of the subject.

Muscle thickness (MT) of the medial gastrocnemius of the right lower leg as a direct measurement for skeletal muscle hypertrophy was measured using B-mode ultrasound (Mindray DP-50, Mindray Medical International Ltd, Shenzhen, China) with 8.5-MHz linear probe (Mindray 75L53EA, Mindray Medical International Ltd, Shenzhen, China). Ultrasound is considered a valid measure for muscle thickness changes compared to other techniques such as magnetic resonance imaging or computed tomography [12,13]. For imaging, the participants were positioned in a prone position on the examination table, with knees fully extended and feet hanging over the edge of the table. A water-soluble gel was applied and the scanning head was positioned perpendicular to the long axis of the shank at 30% distance between the popliteal crease and the lateral malleolus, at the most prominent point of the muscle belly [14]. Two images were recorded by the same researcher for each subject without depression of the underlying tissue (gain = 50 dB; image depth = 4.6 cm). MT, defined as the distance between the deep and superficial aponeurosis, was measured and the mean value of both measurements was used for further evaluation.

Subsequently, to warm up, participants completed five minutes low-intensity treadmill running. Leg stiffness (LS) was then assessed using the Optojump photocell system (Microgate Srl, Bolzano, Italy) [15]. For the Optojump system a standard error of measurement ranging 1.8–2.1 kN/m has been reported previously [15]. Participants completed three trials of the seven-maximal hopping test, with two minutes of rest between trials. The first trial served as a task specific warm-up; only the second and third trials were documented. Subjects were instructed to jump as high as possible with hands on hips and minimal ground contact time. If subjects failed to realize mean ground contact times under 200ms, the attempt was repeated until two valid trials were obtained, but not more than twice. All trials were performed without shoes or socks on gym flooring. Mean flight and ground contact times from all jumps of a single valid trial, together with subject body mass, were taken to calculate LS using the formula proposed by Ruggiero et al. [15]. Mean values of both trials was documented for further evaluation.

Following five minutes of rest, 1-RM calf raise was conducted using a Smith machine (Life Fitness Inc, Rosemont, Illinois, United States). The participants were positioned with their forefoot on a step and the barbell bar resting on their neck. Maximum ankle plantar flexion with an unloaded bar was measured using a goniometer. Warm-up sets of 10, 5, 3 and 1 repetitions were done using 50, 65, 80 and 90% of the participants estimated 1-RM, interspersed with 1, 2, 3 and 4 minutes of rest respectively [16]. Afterward, 1-RM was determined by increasing resistance until the subjects failed the attempt, with four minutes of rest between trials. Repetitions were only valid if the previously measured range of motion was reached. During all maximal trials, strong verbal encouragement was given by the researcher.

After pre-testing, all subjects completed 2 sets of 10 repetitions, one with and one without BFR, using 30% of their 1-RM to get familiar with both training modalities. For BFR, a practical approach was utilized [17], using 7cm wide cuffs with a twist lock (mybimaxx GmbH, Kirchheim unter Teck, Germany). BFR cuffs were placed below the patella of both legs and tightened to the maximum (until no further turning of the lock was possible) to restrict venous blood flow, and to reduce arterial inflow. Measurements taken before the start of this study showed that this procedure leads a reduction of arterial blood flow of 59.76% (5.75) in young men (for more detail, see the S1 Table). After all pre-tests were completed, participants were randomly assigned to either the BFR (n = 15) or NoBFR (n = 14) intervention group, based on their initial 1-RM.

#### Intervention

The experiment was conducted from October 2018 to December 2018. The intervention lasted six weeks and consisted of one calf exercise on two non-consecutive days of the week (minimum 48 and maximum 96 hours apart) in addition to the subjects’ usual training regimen. All training sessions were supervised by one of the researchers. Following five minutes of low-intensity treadmill running, subjects completed four sets of standing calf raise exercise, as described in the pre-testing section, with 30% of their pre-intervention 1-RM at full ROM to concentric muscle failure, either with or without BFR, according to their assigned group. Concentric muscle failure was defined as the inability to reach full ROM, which was controlled by a researcher during each set using a goniometer. In the BFR group, the cuffs were placed and closed before the first set, as previously described in the pre-testing section, and were not loosened or taken off during the rest periods until the fourth set was completed. Inter set rest periods for both groups were set at 30 seconds. Cadence of every repetition was set as 2-second eccentric and 2-second concentric, with no isometric hold at the top or the bottom of each repetition. This was monitored via mobile application Pro Metronome (Xanin Technology, Berlin, Germany). The number of repetitions of all sets during each training session were documented in a training log. Pain was assessed after every set during the first session of the intervention using Visual Analogue Scale (VAS), as described by Heller et al. [18].

All subjects had to attend at least of nine of the 12 (75% adherence) training sessions to be included for analysis. If participants missed two sessions in a row or more than three total, they were eliminated from the study. During the intervention, eight subjects missed more than three training sessions and were eliminated for further analysis (health reasons n = 5, personal reasons n = 3). None of the subjects were injured during the RT sessions.

*Post-testing*. Post-testing took place at least three days and not more than five days after the last training session. All procedures were identical to pre-testing. All researchers performing assessments were unaware of subjects’ intervention group allocation. Data from 21 subjects finishing the study were included for analysis. See Table 1 for further information on both groups.

**Table 1:** Baseline group characteristics. Values are displayed as a mean (standard deviation).

	BFR (n = 12)	NoBFR (n = 9)
Age (y)	27.33 (7.0)	28.9 (7.4)
Height (cm)	182.3 (5.0)	185.0 (7.8)
Body mass (kg)	82.6 (6.0)	83.9 (11.6)
RT experience (y)	7.3 (7.0)	7.1 (6.6)

RT = resistance training; BFR = blood flow restriction training; NoBFR = no blood flow restriction training.

### Statistical analysis

Statistical analyses were performed using SPSS (version 25; IBM Corp, Chicago, IL). Data were tested for normal distribution using Shapiro–Wilk test and homogeneity of variance using Levene’s test. Data are presented as means (SD). One-way analysis of variance (ANOVA) was used to test for baseline differences between groups. A 2×2 (time × group) ANOVA was used to determine differences in CV, MT, LS, and 1-RM. In addition, a 5x2 (time x group) repeated measures ANOVA was used to analyze the interaction of VAS values across time and group. An independent-samples t-test was conducted to compare the mean number of repetitions completed by the subjects per training session in both groups. A paired t-test was used to detect differences in the total number of repetitions in RT sessions 1 and 11. RT session 12 was not used for analysis as a number of subjects missed this session. Where necessary, Bonferroni post-hoc analysis was performed (adjusted for type I error). Within-group effect sizes (ES) were calculated as suggested by Dankel and Loenneke using the following formula: mean change score / SD of the mean change score [19]. The change score was calculated as the post-test value minus the pre-test value for each participant.

In addition, 95% confidence intervals (95%CI) for mean differences and mean percentage changes were calculated. Statistical significance was defined as p ≤ .05. Intraclass Correlation Coefficients (ICC) estimates were calculated based on a mean-rating (k = 2), absolute-agreement, 2-way random-effects model for MT and LS.

## Results

Mean difference between the 50 repeated measures of MT across all participants, regardless of group assignment, was 0.001cm (0.05), and -0.64 kN/m (2.95) for LS. A high degree of measurement reliability was found. Mean measure ICC was 0.984 for MT, and 0.941 for LS respectively. Participants in the BFR attended a mean of 11.67 (0.49) of 12 possible trainings sessions and in the NoBFR 11.67 (0.50), respectively. Between RT session 1 and 11 the BFR group increased the total number of repetitions across all sets from 46 (7) to 59 (14) (p = 0.006), while in the NoBFR group there was no significant change (p = 0.318; RT session 1: 71 (22); RT session 2: 79 (19). The mean number of repetitions completed per training session was significantly higher in the NoBFR group (70 (16)) compared to the BFR group (52 (9)) (p = 0.002).

**Fig 1:**
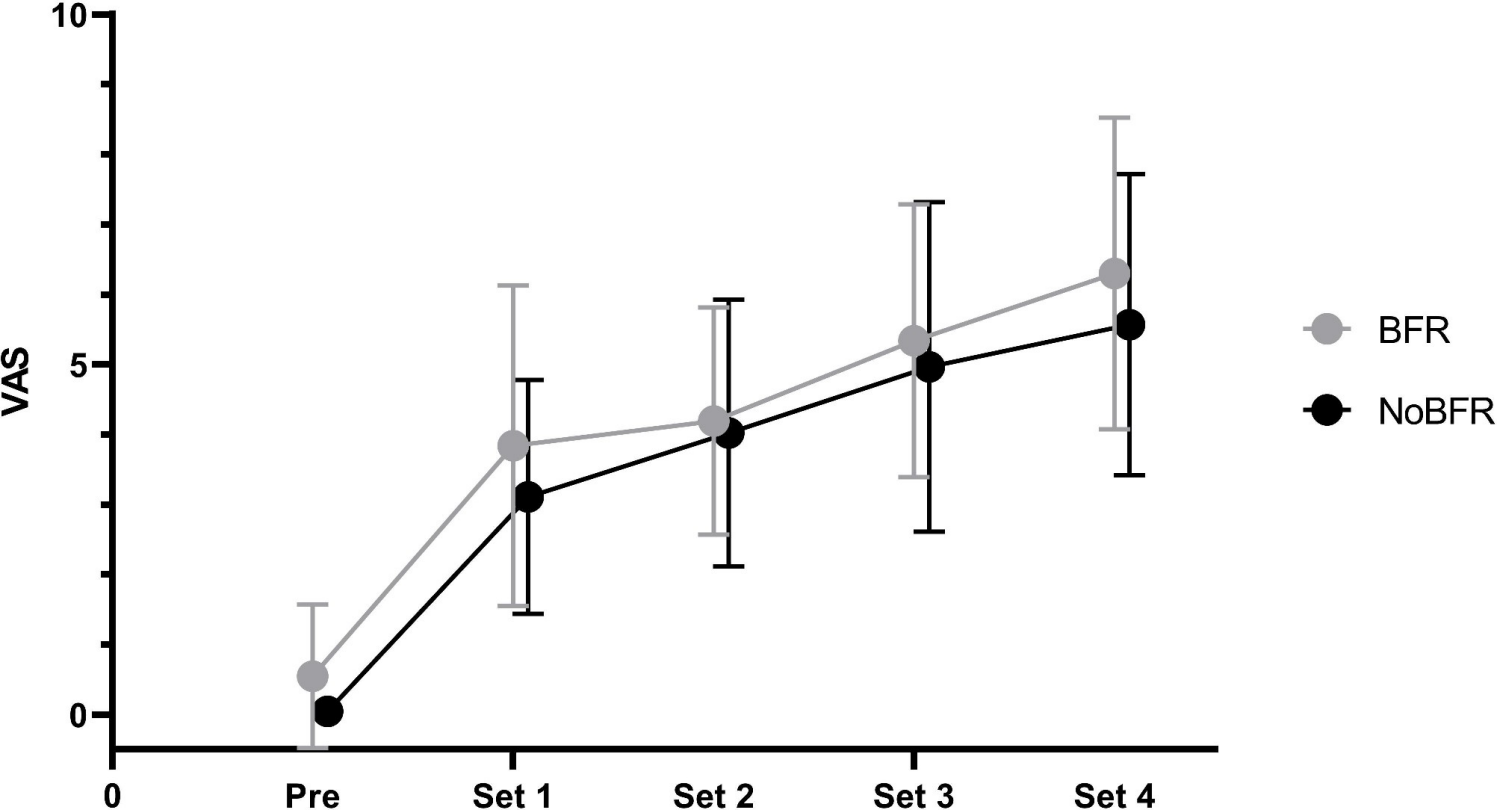
Mean values of pain perception using a Visual Analogue Scale (VAS) from pre-training across four sets of low intensity blood flow restriction (BFR) or low-intensity conventional calf muscle training (NoBFR) until failure. Error bars represent standard deviations.

There was no time x group interaction (p = 0.834), nor group effect (p = 0.447) in VAS. However, a time effect was observed (p<0.001) (see Fig 1). VAS in the BFR group following sets 1, 2, 3, and 4 was 0.6 (1.0), 3.8 (2.3), 5.3 (2.0), and 6.3 (2.2) respectively. Subjects of the NoBFR group reported VAS values of 0.0 (0.1), 3.1 (1.7), 4.0 (1.9), and 5.6 (2.2).

**Fig 2:**
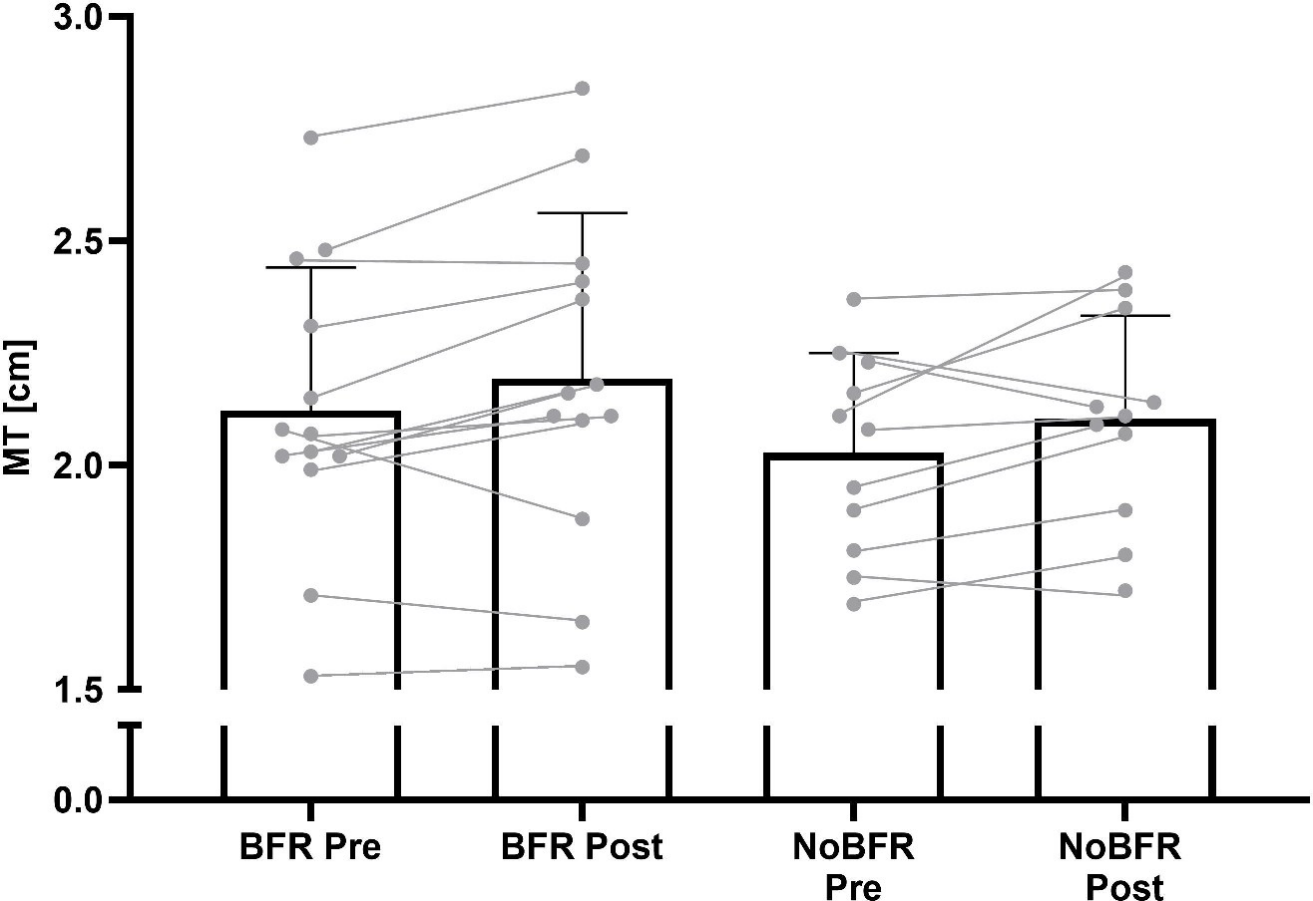
Mean and individual changes of M. gastrocnemius muscle thickness pre and post blood flow restriction (BFR) or unrestricted resistance training (NoBFR). Error bars represent standard deviations.

All other results are summarized in Table 2. There were no significant group differences for any measurement at baseline. No time×group interactions were found. Further analysis showed no group (CV, MT, LS, 1-RM), but significant time effects for MT and 1-RM. The BFR group increased MT from 2.13cm (0.33) to 2.20cm (0.39). This is a mean increase of 0.07cm (ES = 0.61; 95% CI: 0.00–0.14 cm). In comparison, there was a meanch change of +0.04cm in the NoBFR group (PRE 2.06cm (0.21); POST 2.10cm (0.20); ES = 0.40; 95%CI: -0.03–0.11 cm). Mean and individual changes can be seen in Fig 2.

**Table 2:** Pre-/Post-Test measurements, relative change, effect sizes, as well as group by time interactions, group, and time effects. Values are displayed as a mean (standard deviation).

	Group	Pre	Post	Mean change score	ES	group * time	group	time
CV [ml]	BFR	3526 (572)	3552 (594)	26 (86)	0.31	0.138	0.686	0.883
	NoBFR	3658 (604)	3631 (564)	-28 (80)	-0.35			
MT [cm]	BFR	2.13 (0.33)	2.20 (0.39)	0.07 (0.12)	0.61	0.926	0.530	0.008**
	NoBFR	2.06 (0.21)	2.10 (0.20)	0.04 (0.11)	0.40			
LS [kNn/m]	BFR	37.42 (5.80)	35.20 (4.52)	-2.21 (5.01)	-0.44	0.201	0.154	0.165
	NoBFR	32.49 (6.64)	32.64 (6.60)	0.15 (3.82)	0.04			
1-RM [kg]	BFR	163 (37)	203 (42)	40 (22)	1.80	0.428	0.852	< 0.001***
	NoBFR	163 (34)	197 (34)	34 (14)	2.50			

CV = calf volume; MT = muscle thickness; LS = leg stiffness; 1-RM = one-repetition maximum; BFR = blood flow restriction training; NoBFR = no blood flow restriction training.

**p<0.01,

***p<0.001

**Fig 3:**
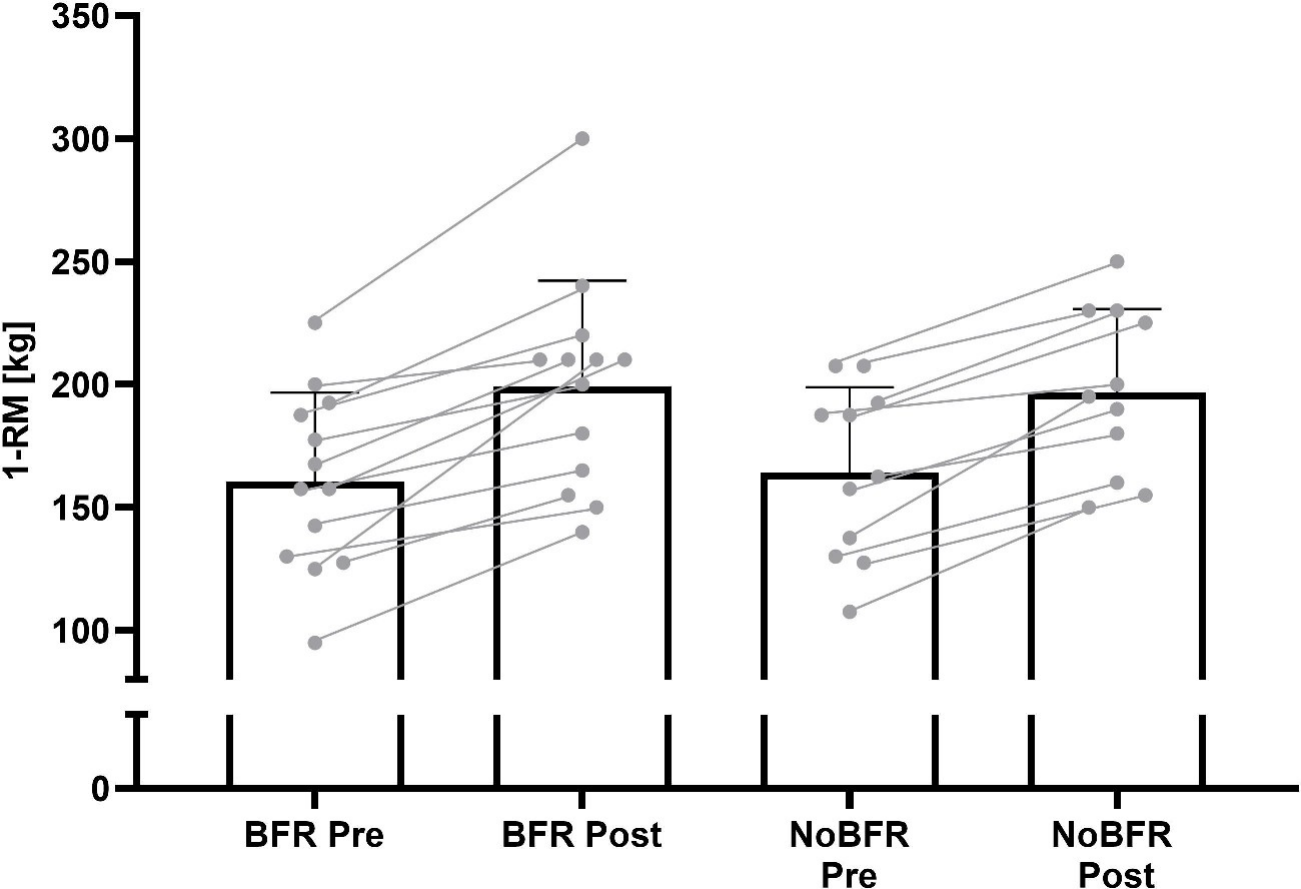
Mean and individual changes of one-repetition maximum standing calf raise pre- and post blood flow restriction (BFR) or unrestricted resistance training (NoBFR). Error bars represent standard deviations.

The BFR group improved their 1-RM from 163kg (37) to 203kg (42), and the NoBFR group from 163 (34) to 197kg (34). This equals a mean 1-RM increase of 40kg in the BFR (ES = 1.80; 95%CI: 28–53 kg), and 34kg in the NoBFR group respectively (ES = 2.50; 95%CI: 28–46 kg). Mean and individual changes can be found in Fig 3.

## Discussion

The aim of this study was to investigate whether low-intensity RT with BFR is superior to RT without BFR to increase calf muscle strength and thickness in trained subjects. We could demonstrate no difference between training groups. In BFR and NoBFR, changes in 1-RM and MT were evident. This study demonstrates that the calf muscles are, in fact, trainable in terms of strength and hypertrophy.

Significant increases in 1-RM were visible in both groups (BFR ES = 1.80 NoBFR ES = 2.50) of 24.80% in the BFR and 20.95% in the NoBFR group, respectively. There are studies showing a lower increases of knee extensor strength through comparable low-load BFR RT protocols (30% 1-RM) to failure. For example Clark et al. [20] and Ellefsen et al. [21] showed an increase in 1-RM of only 8% in four weeks of RT and 10% after twelve weeks respectively. Only studies using protocols not to failure showed comparably high results of 19% [22] and 21% strength gain [23] respectively. However, it should be noted that different protocols (e.g. repetition matched protocols, or protocols to failure) and exercises (e.g. knee extension, leg press) lead to different adaptations; it is important to be aware of this when comparing previous studies to the results of the RT protocol for the calves used in this study. Nonetheless, the results in this study are higher than the ES of low-load BFR RT for muscles other than the calves, described in a meta-analysis by Loenneke et al. [24]. However, comparisons of ES must be done with extreme caution, since researchers use different types of ES or do not provide precise information on how they were calculated [25]. Whether the increase in strength resulted primarily from structural rather than neuronal adaptations requires further investigation. In general, it has been suggested that higher strength gains are expected when higher intensities, frequencies, and longer BFR RT interventions are used [6]. Therefore, studies comparing different modalities and combinations of intensities (e.g. high-load RT vs. low-load BFR RT, high-load RT vs. high-load BFR RT) are needed.

Low-intensity BFR and NoBFR resulted in changes of MT, even after only six weeks (BFR ES = 0.61; NoBFR ES = 0.40). The relative change mean change was 3.29% gain in BFR and 1.94% in NoBFR group. Other studies on hypertrophy following low-intensity BFR RT (20–30% 1-RM) of the quadriceps showed slightly higher results (between 6 and 7%) [26,21,27]. However, the duration of the RT periods were substantially longer these studies (8–12 weeks), while lower intensities were used compared to this study. Furthermore, only Ellefsen et al. [21] used a RT protocol until failure. Therefore, it is difficult to compare these results. Concerning calf RT in general, it is also difficult to compare results, since studies used a variety of training methods, as well as different repetitions schemes, intensities, sets, and intervention durations. used For example, Weiss et al. [28] used four sets of nine to 13 repetitions thrice per week over eight weeks, while subjects by Kubo et al. [29] trained with five sets of ten repetitions at 80% of 1-RM four times a week over twelve weeks. Nonetheless, it is remarkable that in this study muscular growth could be detected after only six weeks of RT. However, since Slysz et al. [6] reported higher MT gains in studies lasting eight weeks or longer, more studies on calf RT with longer intervention periods are needed.

CV remained unchanged in both groups. This is surprising, as significant changes in MT were present. It is also worth noting that CV changes were smaller than the usual measurement error of the device [11]. This could explain the present discrepancy of the results. To our knowledge, the Perometer has never been used to determine muscle growth. Therefore, studies evaluating the suitability of the Perometer for this purpose are desirable, since the measurement takes only a few seconds and could be a valuable addition to existing methods, such as ultrasound.

Tests of LS are a commonly used procedure to characterize stretch-shortening cycle (SSC) function [30], which plays a vital role in athletic tasks like sprinting or jumping. And, since LS depends primarily on ankle stiffness [31], and ankle stiffness depends in part on the properties of the Achilles tendon [32], the seven-maximal hopping test was selected to indirectly obtain information about the adaptations of the Achilles tendon to low-intensity BFR or NoBFR RT. Although the Optojump device underestimates LS compared to the gold standard (3D motion capture system in combination with a force plate) it is considered a reliable field test for LS [15]. However, our results show no differences in LS after the intervention. This is in line with previous research, showing that low-load BFR training was not sufficient to elict changes in tendon stiffness [26]. Therefore, for athletes who do not only want to gain MT and strength through RT, low-intensity BFR training was considered not ideal, because the disproportional increase in strength through BFR RT with little or no adaptations of the tendon might lead to an unfavourable imbalance of force-generating capacity and the mechanical properties of the tendon, which is considered a risk factor for tendinopathies [33].

However, in contrast to Kubo et al. [26], more recent research found comparable increments in Achilles tendon stiffness and cross-sectional area after low-load BFR RT (20–35% 1-RM) compared to a high load protocol (70–85% 1-RM) [34]. This is surprising, since it was previously thought, that concerning tendon adaptations in general, higher load RT is more effective than low-intensity RT to increase tendon stiffness. Besides, literature suggests that 12 weeks or more are required for tendon adaptations to take place [35]. This could explain why Centner et al. [34] with their study spanning 14 weeks were able to show differences in tendon stiffness, while the present study only lasting six weeks could not. It should also be noted that the test for LS used in the present study only allows indirect conclusions to be drawn on the Achilles tendon and that changes in LS (-2.21 kN/m BFR; 0.15 kN/m NoBFR) were approximately in the range of the measurement error of 1.8–2.1 kN/m reported previously [15]. Therefore, more studies on tendon adaptations following long-term low-intensity BFR RT, measured by direct techniques such as ultrasound, are needed.

VAS of pain increased significantly in both groups from baseline to the fourth set. This is in line with previous research [36]. There may be however differences when comparing low-intensity BFR with high-intensity NoBFR RT or RT which is not performed to muscle failure [37]. However, this too requires further investigation.

The number of completed repetitions in a single session increased in BFR group over time (from 46 (7) to 59 (14)). It can be assumed, that through higher repetition volume, the principle of progressive overload was respected and lead to a sufficient training stimulus, even after six weeks. However, in the NoBFR group there was only a trend towards higher repetitions per session (71 (22) to 79 (19)). Nonetheless, adaptations were fairly identical in both groups. Whether differences would be present after longer intervention periods needs further research. The mean number of repetitions completed by the subjects per training session was significantly higher in the NoBFR group (70 (16) vs. 52 (9)), but in the usual range of this type of training [38]. This is in line with previous work, showing lower repetition volume under BFR conditions in comparison to NoBFR RT [39,40].

One strong point of this study is the inclusion of RT-trained subjects only. This makes the results more applicable for populations with longer RT backgrounds. However, trained subjects have less potential for adaptation [41]. Nevertheless, this underlines the importance of our results, as even trained participants showed improvements in calf strength and MT through low-intensity RT, regardless of with or without BFR. It must be mentioned that there was no time matched non-exercise control group, especially since subjects were allowed to perform additional training. This may have influenced the adaptation of the calves as well. It is therefore possible that the resulting systemic release of hormones and growth factors led to an additional increase in MT [42]. However, in a recent review on the role of hormones in RT, the authors conclude that acute hormonal elevations may at best have minor impact on hypertrophy [43]. In the authors’ opinion, a complete cessation of subjects’ additional training would have been a greater source of bias due to detraining effects. In addition, it would be far from normal training practice to conduct studies in which only calves are allowed to be trained. It should also be mentioned that dietary habits were not regulated or documented. Since nutrition can have an influence on muscle growth, future studies should consider controlling the diet. Another limitation may be the BFR cuffs used. The current literature recommends 50–80% of the pressure required to completely occlude arterial flow during low-intensity BFR training [38]. The cuffs used in this study induced a mean of 59.76% (5.75) reduction of arterial blood flow, which is in line with the aforementioned recommendation. Therefore, the cuffs used in this study seem appropriate for BFR RT and more practical compared to other more expensive specialized pressure devices.

In conclusion, BFR and NoBFR RT lead to improvements of 1-RM and MT in RT trained males. LS and CV, however, remained unchanged. VAS values of pain in both groups were similar. For these reasons, it can be stated that BFR calf training is superior to NoBFR RT, as it leads to similar results but with less time, due to less repetitions required for completing a set until concentric muscle failure. Future studies should consider younger and older participants, trained and untrained, as well as women of all ages. In addition, researchers should consider using a time matched control group, longer intervention periods with different RT frequencies and training intensities. Further research on on RT periodization strategies, which include traditional high-intensity RT as well as low-load BFR RT on measures of strength, hypertrophy and athletic performance would also be a valuable contribution on the subject.

## Supporting information

S1 Table Mean reduction in arterial blood flow velocity of the arteria tibialis anterior with or without a BFR cuff in healthy young men (age 29 (3.05) years). Four heartbeats were used to determine TA mean by Doppler ultrasound (SonoSCape P20 with SonoScape L742 probe, SonoScape Europe S.r.l., Rome, Italy). Each condition was measured three times and the mean was calculated. The 7cm wide BFR cuff with a twist lock (mybimaxx GmbH, Kirchheim unter Teck, Germany) was placed below the patella of the left leg and tightened to the maximum. The difference between “TA mean no cuff” and “TA mean with cuff” in percent equaled the resulting reduction in arterial blood flow velocity. BFR = blood flow restriction training; TA = Time averaged arterial blood flow velocity.
(XLSX)

## References

[1] Möck S, Hartmann R, Wirth K, Rosenkranz G, Mickel C (2018) Correlation of dynamic strength in the standing calf raise with sprinting performance in consecutive sections up to 30 meters. Research in sports medicine 26 (4): 474–481. 10.1080/15438627.2018.1492397 29963928

[2] Rumpf MC, Lockie RG, Cronin JB, Jalilvand F (2016) Effect of Different Sprint Training Methods on Sprint Performance Over Various Distances. A Brief Review. Journal of strength and conditioning research 30 (6): 1767–1785. 10.1519/JSC.0000000000001245 26492101

[3] Trappe TA, Raue U, Tesch PA (2004) Human soleus muscle protein synthesis following resistance exercise. Acta physiologica Scandinavica 182 (2): 189–196. 10.1111/j.1365-201X.2004.01348.x 15450115

[4] Behringer M, Moser M, Montag J, McCourt M, Tenner D et al (2015) Electrically induced muscle cramps induce hypertrophy of calf muscles in healthy adults. Journal of musculoskeletal & neuronal interactions 15 (2): 227–236. 26032216 PMC5133727

[5] Schoenfeld BJ (2010) The mechanisms of muscle hypertrophy and their application to resistance training. Journal of strength and conditioning research 24 (10): 2857–2872. 10.1519/JSC.0b013e3181e840f3 20847704

[6] Slysz J, Stultz J, Burr JF (2016) The efficacy of blood flow restricted exercise. A systematic review & meta-analysis. Journal of science and medicine in sport 19 (8): 669–675. 10.1016/j.jsams.2015.09.005 26463594

[7] Lixandrão ME, Ugrinowitsch C, Berton R, Vechin FC, Conceição MS et al (2018) Magnitude of Muscle Strength and Mass Adaptations Between High-Load Resistance Training Versus Low-Load Resistance Training Associated with Blood-Flow Restriction. A Systematic Review and Meta-Analysis. Sports medicine 48 (2): 361–378. 10.1007/s40279-017-0795-y 29043659

[8] Evans C, Vance S, Brown M (2010) Short-term resistance training with blood flow restriction enhances microvascular filtration capacity of human calf muscles. Journal of sports sciences 28 (9): 999–1007. 10.1080/02640414.2010.485647 20544482

[9] Bunevicius K, Grunovas A, Trinkunas E, Poderienė K, Silinskas V et al (2019) Low- and high-intensity one-week occlusion training improves muscle oxygen consumption and reduces muscle fatigue. The Journal of sports medicine and physical fitness 59 (6): 941–946. 10.23736/S0022-4707.18.08672-3 29991216

[10] Faul F, Erdfelder E, Buchner A, Lang A-G (2009) Statistical power analyses using G*Power 3.1. Tests for correlation and regression analyses. Behavior research methods 41 (4): 1149–1160. 10.3758/BRM.41.4.1149 19897823

[11] Man IOW, Markland KL, Morrissey MC (2004) The validity and reliability of the Perometer in evaluating human knee volume. Clinical physiology and functional imaging 24 (6): 352–358. 10.1111/j.1475-097X.2004.00577.x 15522044

[12] Franchi MV, Longo S, Mallinson J, Quinlan JI, Taylor T et al (2018) Muscle thickness correlates to muscle cross-sectional area in the assessment of strength training-induced hypertrophy. Scandinavian journal of medicine & science in sports 28 (3): 846–853. 28805932 10.1111/sms.12961 PMC5873262

[13] Loenneke JP, Dankel SJ, Bell ZW, Spitz RW, Abe T et al (2019) Ultrasound and MRI measured changes in muscle mass gives different estimates but similar conclusions. A Bayesian approach. European journal of clinical nutrition 73 (8): 1203–1205. 10.1038/s41430-019-0431-z 31015562

[14] Legerlotz K, Smith HK, Hing WA (2010) Variation and reliability of ultrasonographic quantification of the architecture of the medial gastrocnemius muscle in young children. Clinical physiology and functional imaging 30 (3): 198–205. 10.1111/j.1475-097X.2010.00925.x 20184623

[15] Ruggiero L, Dewhurst S, Bampouras TM (2016) Validity and Reliability of Two Field-Based Leg Stiffness Devices. Implications for Practical Use. Journal of applied biomechanics 32 (4): 415–419. 10.1123/jab.2015-0297 26959196

[16] Gavanda S, Geisler S, Quittmann OJ, Schiffer T (2019) The Effect of Block Versus Daily Undulating Periodization on Strength and Performance in Adolescent Football Players. International journal of sports physiology and performance 14 (6): 814–821. 10.1123/ijspp.2018-0609 30569761

[17] Lowery RP, Joy JM, Loenneke JP, Souza EO de, Machado M et al (2014) Practical blood flow restriction training increases muscle hypertrophy during a periodized resistance training programme. Clinical physiology and functional imaging 34 (4): 317–321. 10.1111/cpf.12099 24188499

[18] Heller GZ, Manuguerra M, Chow R (2016) How to analyze the Visual Analogue Scale. Myths, truths and clinical relevance. Scandinavian journal of pain 13: 67–75. 10.1016/j.sjpain.2016.06.012 28850536

[19] Dankel SJ, Loenneke JP (2018) Effect Sizes for Paired Data Should Use the Change Score Variability Rather Than the Pre-test Variability. Journal of strength and conditioning research. 10.1519/JSC.0000000000002946 30358698

[20] Clark BC, Manini TM, Hoffman RL, Williams PS, Guiler MK et al (2011) Relative safety of 4 weeks of blood flow-restricted resistance exercise in young, healthy adults. Scandinavian journal of medicine & science in sports 21 (5): 653–662. 21917016 10.1111/j.1600-0838.2010.01100.x PMC6152804

[21] Ellefsen S, Hammarström D, Strand TA, Zacharoff E, Whist JE et al (2015) Blood flow-restricted strength training displays high functional and biological efficacy in women. A within-subject comparison with high-load strength training. American journal of physiology. Regulatory, integrative and comparative physiology 309 (7): R767–79. 10.1152/ajpregu.00497.2014 26202071 PMC4666930

[22] Karabulut M, Abe T, Sato Y, Bemben MG (2010) The effects of low-intensity resistance training with vascular restriction on leg muscle strength in older men. European journal of applied physiology 108 (1): 147–155. 10.1007/s00421-009-1204-5 19760431

[23] Libardi CA, Chacon-Mikahil MPT, Cavaglieri CR, Tricoli V, Roschel H et al (2015) Effect of concurrent training with blood flow restriction in the elderly. International journal of sports medicine 36 (5): 395–399. 10.1055/s-0034-1390496 25700103

[24] Loenneke JP, Wilson JM, Marín PJ, Zourdos MC, Bemben MG (2012) Low intensity blood flow restriction training. A meta-analysis. European journal of applied physiology 112 (5): 1849–1859. 10.1007/s00421-011-2167-x 21922259

[25] Kelley K, Preacher KJ (2012) On effect size. Psychological methods 17 (2): 137–152. 10.1037/a0028086 22545595

[26] Kubo K, Komuro T, Ishiguro N, Tsunoda N, Sato Y et al (2006) Effects of low-load resistance training with vascular occlusion on the mechanical properties of muscle and tendon. Journal of applied biomechanics 22 (2): 112–119. 10.1123/jab.22.2.112 16871002

[27] Laurentino GC, Ugrinowitsch C, Roschel H, Aoki MS, Soares AG et al (2012) Strength training with blood flow restriction diminishes myostatin gene expression. Medicine and science in sports and exercise 44 (3): 406–412. 10.1249/MSS.0b013e318233b4bc 21900845

[28] Weiss LW, Clark FC, Howard DG (1988) Effects of heavy-resistance triceps surae muscle training on strength and muscularity of men and women. Physical therapy 68 (2): 208–213. 10.1093/ptj/68.2.208 3340658

[29] Kubo K, Morimoto M, Komuro T, Yata H, Tsunoda N et al (2007) Effects of plyometric and weight training on muscle-tendon complex and jump performance. Medicine and science in sports and exercise 39 (10): 1801–1810. 10.1249/mss.0b013e31813e630a 17909408

[30] Ste Croix MBA de, Hughes JD, Lloyd RS, Oliver JL, Read PJ (2017) Leg Stiffness in Female Soccer Players. Intersession Reliability and the Fatiguing Effects of Soccer-Specific Exercise. Journal of strength and conditioning research 31 (11): 3052–3058. 10.1519/JSC.0000000000001715 29065079

[31] Farley CT, Morgenroth DC (1999) Leg stiffness primarily depends on ankle stiffness during human hopping. Journal of biomechanics 32 (3): 267–273. 10.1016/s0021-9290(98)00170-5 10093026

[32] Wikstrom EA, Tillman MD, Chmielewski TL, Borsa PA (2006) Measurement and evaluation of dynamic joint stability of the knee and ankle after injury. Sports medicine 36 (5): 393–410. 10.2165/00007256-200636050-00003 16646628

[33] Mersmann F, Bohm S, Arampatzis A (2017) Imbalances in the Development of Muscle and Tendon as Risk Factor for Tendinopathies in Youth Athletes. A Review of Current Evidence and Concepts of Prevention. Frontiers in physiology 8: 987 10.3389/fphys.2017.00987 29249987 PMC5717808

[34] Centner C, Lauber B, Seynnes OR, Jerger S, Sohnius T et al (2019) Low-load blood flow restriction training induces similar morphological and mechanical Achilles tendon adaptations compared with high-load resistance training. Journal of applied physiology (Bethesda, Md.: 1985) 127 (6): 1660–1667. 10.1152/japplphysiol.00602.2019 31725362

[35] Bohm S, Mersmann F, Arampatzis A (2015) Human tendon adaptation in response to mechanical loading. A systematic review and meta-analysis of exercise intervention studies on healthy adults. Sports medicine—open 1 (1): 7 10.1186/s40798-015-0009-9 27747846 PMC4532714

[36] Wernbom M, Järrebring R, Andreasson MA, Augustsson J (2009) Acute effects of blood flow restriction on muscle activity and endurance during fatiguing dynamic knee extensions at low load. Journal of strength and conditioning research 23 (8): 2389–2395. 10.1519/JSC.0b013e3181bc1c2a 19826283

[37] Loenneke JP, Kim D, Fahs CA, Thiebaud RS, Abe T et al (2015) The effects of resistance exercise with and without different degrees of blood-flow restriction on perceptual responses. Journal of sports sciences 33 (14): 1472–1479. 10.1080/02640414.2014.992036 25555163

[38] Scott BR, Loenneke JP, Slattery KM, Dascombe BJ (2015) Exercise with blood flow restriction. An updated evidence-based approach for enhanced muscular development. Sports medicine 45 (3): 313–325. 10.1007/s40279-014-0288-1 25430600

[39] Fahs CA, Loenneke JP, Thiebaud RS, Rossow LM, Kim D et al (2015) Muscular adaptations to fatiguing exercise with and without blood flow restriction. Clinical physiology and functional imaging 35 (3): 167–176. 10.1111/cpf.12141 24612120

[40] Farup J, Paoli F de, Bjerg K, Riis S, Ringgard S et al (2015) Blood flow restricted and traditional resistance training performed to fatigue produce equal muscle hypertrophy. Scandinavian journal of medicine & science in sports 25 (6): 754–763. 25603897 10.1111/sms.12396

[41] Williams TD, Tolusso DV, Fedewa MV, Esco MR (2017) Comparison of Periodized and Non-Periodized Resistance Training on Maximal Strength. A Meta-Analysis. Sports medicine 47 (10): 2083–2100. 10.1007/s40279-017-0734-y 28497285

[42] Kraemer WJ, Ratamess NA (2005) Hormonal responses and adaptations to resistance exercise and training. Sports medicine 35 (4): 339–361. 10.2165/00007256-200535040-00004 15831061

[43] Fink J, Schoenfeld BJ, Nakazato K (2018) The role of hormones in muscle hypertrophy. The Physician and sportsmedicine 46 (1): 129–134. 10.1080/00913847.2018.1406778 29172848

